# Response surface methodological optimization of l-asparaginase production from the medicinal plant endophyte *Acinetobacter baumannii* ZAS1

**DOI:** 10.1186/s43141-022-00309-4

**Published:** 2022-02-09

**Authors:** K. N. Abhini, Akhila B. Rajan, K. Fathimathu Zuhara, Denoj Sebastian

**Affiliations:** Department of Life Sciences, University of Calicut, Malappuram, Kerala 673635 India

**Keywords:** *Acinetobacter baumannii*, *Annona muricata*, Central composite design, Endophyte, l-asparaginase, Response surface methodology

## Abstract

**Background:**

This study targets the enhanced production of l-asparaginase, an antitumor enzyme by *Acinetobacter baumannii* ZAS1. This organism is an endophyte isolated from the medicinal plant *Annona muricata*. Plackett–Burman design (PBD) and central composite design (CCD) were used for statistical optimization of media components.

**Results:**

The organism exhibited 18.85 ± 0.2 U/mL enzyme activities in unoptimized media. Eight variables: l-asparagine, peptone, glucose, lactose, yeast extract, NaCl, MgSO_4_, and Na_2_HPO_4_ were screened by PBD. Among them, only four factors—l-asparagine, peptone, glucose, and Na_2_HPO_4_—were found to affect enzyme production significantly (*p* < 0.05). Furthermore, the best possible concentrations and interactive effects of the components that enhance this enzyme's output were chosen by using CCD on these selected variables. The results revealed that an optimized medium produces a higher concentration of enzymes than the unoptimized medium. After optimizing media components, the maximum l-asparaginase activity was 45.59 ± 0.36 U/mL, around the anticipated value of 45.04 ± 0.42 U/mL. After optimization of process parameters, it showed a 2.41-fold increase in the production of l-asparaginase by the endophyte *Acinetobacter baumannii* ZAS1.

**Conclusion:**

The findings of this study indicated that an endophyte, *Acinetobacter baumannii* ZAS1 that produces l-asparaginase could be used to increase enzyme output. However, using the statistical methods Plackett-Burman design and central composite design of response surface methodology is a handy tool for optimizing media components for increased l-asparaginase synthesis.

**Supplementary Information:**

The online version contains supplementary material available at 10.1186/s43141-022-00309-4.

## Background

l-asparaginase (EC 3.5.1.1) is an enzyme that belongs to the amidase group, which catalyzes the conversion of l-asparagine into aspartic acid and ammonia [1]. The amino acid l-asparagine is essential for cell survival and is primarily involved in the synthesis of several essential proteins [2]. It is a non-essential amino acid that healthy cells can make with the help of the enzyme l-asparagine synthetase [3]. However, due to the insufficient expression of this particular enzyme in certain tumors, such as leukemic cells, they rely on the extracellular supply of this amino acid for their multiplication and survivability [3]. This suggests the importance of l-asparagine deprivation therapy for the selective elimination of tumor cells. l-asparaginase is one of the drugs used in the treatment of acute lymphoblastic leukemia (ALL).

The commercially available l-asparaginase is obtained from bacteria, mainly *Escherichia coli* and *Erwinia chrysanthemi*. This enzyme derived from *Escherichia coli* exhibits several side effects when used as a drug, including a high rate of hypersensitive reactions [4, 5]. Hypersensitivity reactions can range from moderate allergic reactions to potentially lethal anaphylaxis [6]. In addition to this, several side effects like edema, serum sickness, bronchospasm, urticaria and rash, itching and swelling of extremities, and erythema also have been reported [7]. An enzyme derived from *Erwinia chrysanthemi* is successfully used to reduce hypersensitivity to 12–20%, but this drug has a shorter half-life and lacks complete remission [7, 8]. The essential prerequisites for developing a bio-better drug are obtaining an enzyme from a novel habitat and augmenting its production through process parameter optimization [9]. Many researchers are now using statistical methods to maximize l-asparaginase production by various microorganisms [10]. Increased l-asparaginase production (17.089 U/mL) from halotolerant *Bacillus licheniformis* PPD37 was achieved by optimizing the process parameters through RSM [11]. Another study also reported improved l-asparaginase production from *Pectobacterium carotovorum* using 19.36 U/mL through RSM [12].

Recent research emphasizes the importance of this enzyme as an anticancer agent in various cancer cell lines [13]. This enzyme is also used in the food industry as an acrylamide-reducing agent in starchy food products. The diverse applications and growing needs have evoked scientists to search for efficient l-asparaginase producers from new environments. Recently, the search for metabolites with desirable properties concentrates on organisms with novel biotopes [14]. These endophytes are microbes that grow within plant tissue symbiotically associated with such a biotope. Bioactive substances from endophytes are gaining attention due to their vast diversity, reduced toxicity, and ability to withstand different environmental conditions [15]. Though there have been several studies of l-asparaginase from various microbial sources, the production of this enzyme from medicinal plant endophytes has received less attention. In this context, we emphasize the need to study l-asparaginase from endophytes and improve its synthesis. Moreover, response surface methodology (RSM) is one of the statistical methods that have been successfully used to optimize the growth conditions of organisms to increase overall metabolite and biomass production [16, 17].

*Acinetobacter baumannii* ZAS1 is an endophyte obtained from the medicinal plant *Annona muricata* and thus expected to produce minimal side effects. This work aimed to optimize physical factors and media components for enhanced l-asparaginase synthesis from this organism. We employed the statistical method Plackett-Burman design and response surface methodology’s central composite design to accomplish this. The main objective of this work was to optimize l-asparaginase production using appropriate media components to produce this enzyme at a low cost. The *Acinetobacter baumannii* ZAS1 strain utilized in this investigation produced merely 18.85 ± 0.2 U/mL of l-asparaginase in the unoptimized medium. As a result, statistical optimization of nutritional requirements is being investigated to boost l-asparaginase synthesis by this organism.

## Methods

The process parameters of the endophyte *Acinetobacter baumannii* ZAS1 were optimized through the OFAT (one factor at a time) method as well as statistical methods like Plackett–Burman design (PBD) and central composite design (CCD) of RSM.

### Microbial strain

The l-asparaginase producing bacterial endophyte *Acinetobacter baumannii* ZAS1 (Genbank Accession No. KX186685) [18], isolated from the medicinal plant *Annona muricata* (Accession No. CALI 7006), was used for the study. The organism was sub-cultured in nutrient agar (NA) medium every month and stored at 4 °C.

### Chemicals

l-asparagine used for the study was purchased from Sisco Research Laboratories Pvt Ltd (SRL). Other chemicals (analytical grade) used were obtained from different commercial sources.

### Media

*Acinetobacter baumannii* ZAS1 cultures were grown in unoptimized M9 medium [19], which contained the following components: l-asparagine (10 g/l), KH_2_PO_4_ (3 g/l), Na_2_HPO_4_ (6 g/l), NaCl (0.5 g/l), CaCl_2_.2H_2_O (0.001 g/l), MgSO_4_.7H_2_O (0.12 g/l), and agar (20 g/l). The pH of the medium was 7.0, and the incubation temperature was set as 37 °C.

### Extracellular l-asparaginase production and extraction

The bacterial culture used as primary inoculum was grown overnight in 10 ml of M9 medium at 37 °C in an incubator (shaking). In 50 ml of M9 broth medium, 1% of the overnight grown culture (adjusted to a McFarland standard of 0.5) was used as an inoculum to produce l-asparaginase. Following incubation, the culture was centrifuged at 10,000× *g* for 10 min at 4 °C, and the supernatant was used to determine the enzyme activity.

### l-asparaginase assay

The Nesslerization reaction [20] is a commonly used method for the determination of l-asparaginase activity. The amount of ammonia liberated was determined in this reaction. At 37 °C, one unit of l-asparaginase activity corresponds to the amount of enzyme required to liberate 1 μmol of ammonia per minute.

### Optimization of physical parameters through OFAT method

The OFAT method was used to optimize the temperature, pH, and agitation speed for l-asparaginase production. For optimizing temperature, the unoptimized medium was seeded with inoculum and incubated in a shaking incubator at varying temperatures (27 °C, 32 °C, 37 °C, 42 °C, and 47 °C). The culture was centrifuged after 24 h of incubation, and the cell-free supernatant was used as a crude enzyme for the l-asparaginase assay. For optimizing pH, the unoptimized medium with varying pH (6, 7, 8, 9, and 10) was seeded with inoculum and incubated in a shaking incubator at an optimized temperature. The appropriate agitation speed for the best production of the enzyme was discovered by inoculating the medium (adjusted to optimized pH) and incubating at various agitation speeds (50, 100, 150, and 200) under optimized temperature. The l-asparaginase assay was performed in duplicates with crude enzyme produced from a 24-h culture.

### Selection of carbon-nitrogen and mineral sources through OFAT method

For selecting the best sources of carbon, nitrogen, and ion, the unoptimized M9 medium was supplemented with one of the carbon (sucrose, lactose, maltose, glucose, fructose, and galactose), nitrogen (peptone, beef extract, yeast extract, malt extract, ammonium chloride, and sodium nitrate), or mineral (NaCl, KCl, Na_2_HPO_4_, KH_2_PO_4_, MgSO_4_) sources. It was inoculated with primary culture and incubated under optimized physical conditions.

### Screening of important variables through PBD

Different carbon, nitrogen, and mineral sources were screened using a statistical method called PBD [21]. The statistical software package MINITAB (Release 16, PA, USA) was used to design the experiments under PBD. Eight variables were considered in this study. Among these variables, one is l-asparagine, which is an inducer for the production of the enzyme. The remaining seven variables were sources of carbon, nitrogen, and ions obtained by preliminary screening of different variables through the OFAT method. Table 1 shows the experimental model for the selection of different variables. Each variable was investigated at its low level (− 1) and high level (+ 1).

**Table 1:** Variables and their codes used in Plackett-Burman design

Code	Nutrients (g/l)	Low (−1)	High (+ 1)
A	l-asparagine	2.5	12.5
B	Peptone	1	5
C	Glucose	0.2	1
D	Lactose	0.2	1
E	Yeast extract	1	5
F	NaCl	0.5	2.5
G	MgSO_4_	0.5	2.5
H	Na_2_HPO_4_	0.5	2.5

This PBD is based on the first-order model;

$$Y={\beta}_0+\varSigma {\beta}_i{x}_i\left(i=1,\dots, k\right)$$

where *Y* is l-asparaginase activity (the response), β_0_ is the model intercept, *β*_i_ is the linear coefficient, and *x*_i_ is the level of an independent variable.

The eight variables selected for the experiment were l-asparagine, peptone, glucose, lactose, yeast extract, NaCl, MgSO_4_, and Na_2_HPO_4_. The statistical tool designed 20 experimental runs to screen those variables. The tests were conducted in duplicate, and the calculations were based on the average enzyme activity (Table 2). Based on this, variables with confidence levels equal to or greater than 95% were thought to impact l-asparaginase production significantly.

**Table 2:** Experimental design and results of PBD

Run order	l-asparagine	Peptone	Glucose	Lactose	Yeast extract	NaCl	MgSO_4_	NA_2_HPO_4_	l-asparaginase activity U/mL )
1	− 1	1	1	− 1	1	1	− 1	− 1	6.13 ± 0.43
2	1	1	− 1	− 1	− 1	− 1	1	− 1	17.18 ± 0.21
3	− 1	− 1	− 1	− 1	− 1	− 1	− 1	− 1	1.59 ± 0.34
4	− 1	− 1	1	1	− 1	1	1	− 1	2.22 ± 0.29
5	1	− 1	− 1	1	1	− 1	1	1	16.88 ± 0.61
6	1	1	− 1	1	1	− 1	− 1	− 1	16.22 ± 0.48
7	− 1	− 1	− 1	− 1	1	− 1	1	− 1	2.34 ± 0.37
8	− 1	1	− 1	1	1	1	1	− 1	5.59 ± 0.25
9	1	1	1	− 1	− 1	1	1	− 1	16.06 ± 0.47
10	1	− 1	1	− 1	1	1	1	1	17.68 ± 0.26
11	− 1	1	1	− 1	− 1	− 1	− 1	1	7.93 ± 0.34
12	1	1	1	1	− 1	− 1	1	1	22.34 ± 0.51
13	1	− 1	− 1	− 1	− 1	1	− 1	1	16.61 ± 0.36
14	− 1	1	1	1	1	− 1	− 1	1	10.59 ± 0.42
15	1	1	− 1	− 1	1	1	− 1	1	16.84 ± 0.33
16	− 1	− 1	1	− 1	1	− 1	1	1	11.5 ± 0.28
17	1	− 1	1	1	− 1	− 1	− 1	− 1	15.61 ± 0.43
18	− 1	− 1	− 1	1	− 1	1	− 1	1	2.38 ± 0.31
19	− 1	1	− 1	1	− 1	1	1	1	9.97 ± 0.51
20	1	− 1	1	1	1	1	− 1	− 1	18.59 ± 0.60

### Optimization of critical medium components using CCD

Variables selected through PBD were subjected to CCD as it comprises duplication of the central point. The CCD containing four variables at its five coded levels (− α, − 1, 0, + 1 and + α) were generated using the statistical software package “Design Expert 7® (Stat-Ease Inc., USA) (Table 3). A total of 30 experimental runs were created (Table 4), in which 24 runs were the blend of actual levels of the study parameters, and the remaining six runs were the replication of medial points. Experimental runs performed at the medial points were to build the curvature and balance the lack of fit values, which describes the significance of the model [22]. CCD based designs are widely used for the production optimization of many industrial enzymes [23, 24]. The experiments were conducted in duplicates and the average l-asparaginase enzyme activity was used to calculate the response.

**Table 3:** Experimental range and levels of independent variables used for CCD

Variables	Components	− α	− 1	0	+ 1	+ α
A	l-asparagine	0	5	10	15	20
B	Peptone	1	3	5	7	9
C	Glucose	0	0.5	1	1.5	2
D	Na_2_HPO_4_	2	4	6	8	10

**Table 4:** CCD of selected variables with the experimental and predicted response

Run	A: l-asparagine		B: peptone		C: glucose		D: Na_2_HPO_4_		l-asparaginase activity (U/mL)		Residuals
	Coded	Actual	Coded	Actual	Coded	Actual	Coded	Actual	Observed	Predicted	
1	0	10	0	5	0	1	0	6	40.3819	42.7545	− 2.37
2	− 1	5	+ 1	7	+ 1	1.5	+ 1	8	29.2708	29.7992	− 0.53
3	− 1	5	+ 1	7	− 1	0.5	+ 1	8	24.7569	24.6849	0.07
4	+ α	20	0	5	0	1	0	6	44.2014	43.2235	0.98
5	− 1	5	− 1	3	+ 1	1.5	+ 1	8	23.7153	23.0642	0.65
6	0	10	0	5	+ α	2	0	6	32.3958	31.911	0.48
7	0	10	0	5	0	1	0	6	43.1597	42.7545	0.41
8	+ 1	15	− 1	3	+ 1	1.5	+ 1	8	38.9931	39.7518	− 0.76
9	+ 1	15	− 1	3	− 1	0.5	− 1	4	28.2292	28.2645	− 0.04
10	0	10	0	5	0	1	− α	2	21.2847	22.3334	− 1.05
11	0	10	0	5	0	1	0	6	42.8125	42.7545	0.06
12	+ 1	15	− 1	3	− 1	0.5	+ 1	8	37.6042	37.5889	0.02
13	0	10	0	5	0	1	+ α	10	37.2569	36.2513	1.01
14	+ 1	15	+ 1	7	− 1	0.5	− 1	4	28.75	28.793	− 0.04
15	− 1	5	− 1	3	− 1	0.5	+ 1	8	19.5486	19.7294	− 0.18
16	− 1	5	+ 1	7	− 1	0.5	− 1	4	16.4236	16.2285	0.20
17	− 1	5	+ 1	7	+ 1	1.5	− 1	4	25.7986	25.2057	0.59
18	+ 1	15	+ 1	7	+ 1	1.5	+ 1	8	40.3819	41.2351	− 0.85
19	− 1	5	− 1	3	− 1	0.5	− 1	4	11.9097	10.4484	1.46
20	0	10	0	5	0	1	0	6	44.2014	42.7545	1.45
21	0	10	0	5	0	1	0	6	42.8125	42.7545	0.06
22	0	10	0	5	0	1	0	6	43.1597	42.7545	0.41
23	− α	0	0	5	0	1	0	6	12.9514	13.9715	− 1.02
24	+ 1	15	− 1	3	+ 1	1.5	− 1	4	34.8264	34.2902	0.54
25	+ 1	15	+ 1	7	+ 1	1.5	− 1	4	36.2153	36.5982	− 0.38
26	0	10	0	5	− α	0	0	6	20.2431	20.771	− 0.53
27	− 1	5	− 1	3	+ 1	1.5	− 1	4	17.4653	17.6461	− 0.18
28	0	10	− α	1	0	1	0	6	24.0625	24.7927	− 0.73
29	0	10	+ α	9	0	1	0	6	32.7431	32.0561	0.69
30	+ 1	15	+ 1	7	− 1	0.5	+ 1	8	36.9097	37.2927	− 0.38

Analysis of variance (ANOVA) of data was performed. The response surface regression procedure was used to fit the experimental results by the second-order polynomial equation:

$${\displaystyle \begin{array}{c}\mathrm{Y}=\kern0.5em {\upbeta}_0+{\upbeta}_1\mathrm{A}+{\upbeta}_2\mathrm{B}+{\upbeta}_3\mathrm{C}+{\upbeta}_4\mathrm{D}+{\upbeta}_{11}{\mathrm{A}}^2+{\upbeta}_{22}{\mathrm{B}}^2+{\upbeta}_{33}{\mathrm{C}}^2+\\ {}\kern2.25em {\upbeta}_{44}{\mathrm{D}}^2+{\upbeta}_{12}\mathrm{AB}+{\upbeta}_{13}\mathrm{AC}+{\upbeta}_{14}\mathrm{AD}+{\upbeta}_{23}\mathrm{BC}+{\upbeta}_{24}\mathrm{BD}+{\upbeta}_{34}\mathrm{CD}\end{array}}$$

where *Y* is the predicted l-asparaginase activity (response), *A*, *B*, *C*, and *D* are the independent variables studied, *β*_0_ is intercept, *β*_1_, *β*_2_, *β*_3_, and *β*_4_ are linear coefficients, *β*_11_, *β*_22_, *β*_33_, *β*_44_ are squared coefficients, and *β*_12_, *β*_13_, *β*_14_, *β*_23_, *β*_24_, and *β*_34_ are interaction coefficients.

### Validation of the model

Experiments were conducted to validate the statistical model at its optimal levels of the most significant variables under a predicted set of conditions.

### Analysis of the growth curve

A growth curve analysis was performed to examine the correlation between bacterial growth and l-asparaginase production in the optimized medium. Bacterial growth was monitored using an optical density (OD) at 600 nm, and activity was recorded in U/mL every 2 h.

## Results

### Extracellular l-asparaginase production

According to quantitative analysis using the Nesslerization reaction, the organism *Acinetobacter baumannii* ZAS1 had 18.85 ± 0.2 U/mL enzyme activities in unoptimized media. The high value obtained indicated the release of the maximal quantity of ammonia after the catalytic activity of l-asparaginase on the substrate l-asparagine.

### Optimization of physical parameters for l-asparaginase production

**Fig. 1:**
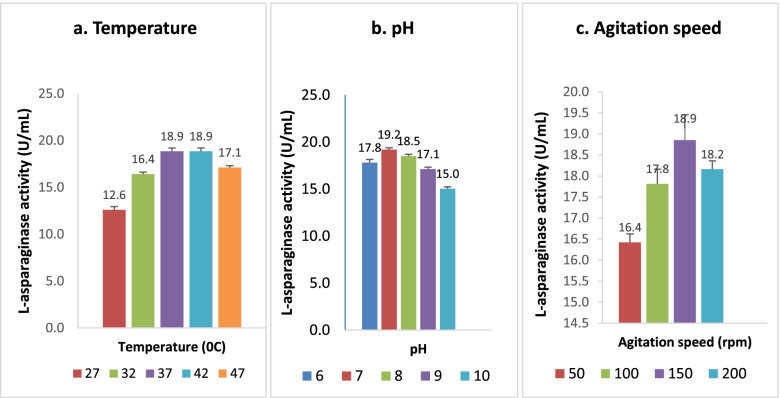
OFAT optimization of **a** temperature, **b** pH, and **c** agitation speed

The physical factors like temperature, pH, and agitation speed that influence l-asparaginase production were optimized by the OFAT method. The results are depicted in Fig. 1. In the case of the temperature study, the endophyte *Acinetobacter baumannii* ZAS1 showed excellent activity (18.854 ± 0.2 U/mL) at 37 °C; this activity was sustained until 42 °C and declined with a further hike in temperature. The optimum pH required for the augmented production (19.201 ± 0.2 U/mL) of l-asparaginase by this organism was pH 7. The pH above and below this point showed decreased activity. Furthermore, the optimum agitation speed required for the maximum production of l-asparaginase enzyme (18.854 ± 0.6 U/mL) by *Acinetobacter baumannii* ZAS1 was 150 rpm. In contrast, the production was lower at speeds above and below this, i.e., at 100 rpm and 200 rpm.

### Primary screening of carbon, nitrogen, and ion sources

**Fig. 2:**
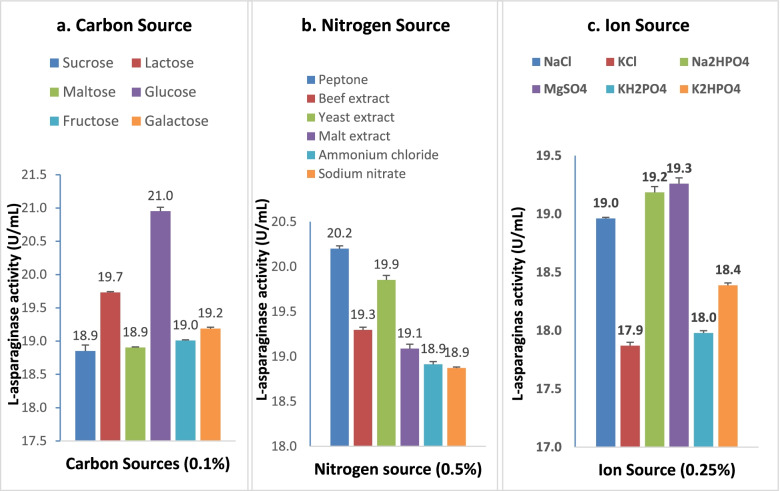
OFAT optimization of **a** carbon, **b** nitrogen, and **c** ion sources supplementing M9 media

The result of the primary screening experiment is shown in Fig. 2. According to the experimental results, glucose and lactose were promising carbon sources for l-asparaginase production among the different carbon sources evaluated (Fig. 2a). Over the various nitrogen sources tested, peptone and yeast extract expressed increased enzyme activity (Fig. 2b). NaCl, Na_2_HPO_4_, and MgSO_4_ showed considerable improvement in the enzyme production from the diverse sources of ions tested (Fig. 2c). According to the findings, the production of l-asparaginase by *Acinetobacter baumannii* ZAS1 improves when different carbon, nitrogen, and ion sources are used in the media.

### Screening of most significant variables through PBD

PBD was applied in this study to determine the main media components influencing l-asparaginase production. The l-asparagine, peptone, glucose, and Na_2_HPO_4_ were found to have a major impact on l-asparaginase synthesis by *Acinetobacter baumannii* ZAS1. The complete experimental design, including response values, is provided in Tables 2 and 5*.* The selected variable, l-asparagine (*p*-value 0), significantly affects the production of l-asparaginase. The second variable selected through the PBD experiment was peptone (*p*-value 0.025) as an additional nitrogen source in the enhanced production of l-asparaginase. The third variable chosen by the PBD experiment was glucose (*p*-value 0.027), which acts as a carbon source for the better production of this enzyme. Through this experiment, the ion source Na_2_HPO_4_ (*p*-value 0.005) also showed a significant effect on l-asparaginase production. The results revealed that additional nitrogen, carbon, and ion sources contributed significantly to the highest production of this enzyme.

**Table 5:** Statistical analysis of PBD of eight variables

Term	Effect	Coef	SE coef	*T*-value	*F*-value	*p*-value	Confidence level (%)
l-asparagine	11.377	5.689	0.452	12.57	158.04	0.000	100^a^
Peptone	2.345	1.172	0.452	2.59	6.71	0.025	97.5^a^
Glucose	2.305	1.152	0.452	2.55	6.49	0.027	97.3^a^
Lactose	0.653	0.327	0.452	0.72	0.52	0.486	51.4
yeast extract	1.047	0.524	0.452	1.16	1.34	0.272	72.8
NaCl	− 1.011	− 0.505	0.452	− 1.12	1.25	0.288	71.2
MgSO4	0.927	0.464	0.452	1.02	1.05	0.328	67.2
Na_2_HPO_4_	3.119	1.56	0.452	3.45	11.88	0.005	99.5^a^
R^2^	0.9445	Adj *R*^2^	0.9042	Pred *R*^2^	0.8166		

^a^Statistically significant at 95% confidence level

### Optimization of important medium components using CCD

In this CCD experiment, the various substances chosen through PBD studies, namely l-asparagine, peptone, glucose, and Na_2_HPO_4_, were treated as four independent variables on which responses were calculated. A total of 30 experimental runs were performed using these four variables. The entire experimental plan, including response values, is described in Table 4.

### Regression analysis

Appropriate conditions for maximum l-asparaginase activity were established using regression analysis. A second-order polynomial equation representing the relationship between enzyme activity, l-asparagine, peptone, glucose, and Na_2_HPO_4_ was generated using multiple regression analyses.

The second-order polynomial equation calculated the predicted l-asparaginase activity is as follows:

$${\displaystyle \begin{array}{c}\mathrm{L}-\mathrm{asparaginase}\ \mathrm{activity}\ \left(\mathrm{U}/\mathrm{mL}\right)=-88.4578+5.0611\mathrm{A}+11.0414\mathrm{B}+43.1387\mathrm{C}\ \\ {}+13.0488\mathrm{D}-0.1312\mathrm{AB}-0.1171\ \mathrm{AC}\ \\ {}+1.08507\mathrm{E}-003\mathrm{AD}+0.4448\mathrm{BC}-0.0515\mathrm{BD}-0.9657\mathrm{C}\mathrm{D}\ \\ {}-0.1415{\mathrm{A}}^2-0.8956{\mathrm{B}}^2-16.4134{\mathrm{C}}^2-0.8413{\mathrm{D}}^2\end{array}}$$

where *A*, *B*, *C*, and *D* are concentrations of l-asparagine, peptone, glucose and Na_2_HPO_4,_ respectively. The *F* test was assessed the statistical significance of the second-order polynomial equation and the results of ANOVA are given in Table 6.

**Table 6:** Analysis of variance (ANOVA) of response surface quadratic model for the production of l-asparaginase

Source	Sum of squares	df	Mean square	*F* value	*p* value prob > *f*	
Model	2918.75	14	208.48	165.72	< 0.0001*	Significant
A—l-asparagine	1283.65	1	1283.65	1020.3	< 0.0001*	
B—Peptone	79.12	1	79.12	62.89	< 0.0001*	
C—Glucose	186.15	1	186.15	147.97	< 0.0001*	
D—Na_2_HPO_4_	290.56	1	290.56	230.96	< 0.0001*	
AB	27.58	1	27.58	21.92	0.0003*	
AC	1.37	1	1.37	1.09	0.3127	
AD	1.884E−0.003	1	1.884E−0.003	1.497E−0.003	0.9696	
BC	37.1	1	3.17	2.52	0.1335	
BD	0.68	1	0.68	0.54	0.4735	
CD	14.92	1	14.92	11.86	0.0036*	
A2	343.56	1	343.56	273.09	< 0.0001*	
B2	352.03	1	352.03	279.83	< 0.0001*	
C2	461.83	1	461.83	367.1	< 0.0001*	
D2	310.68	1	310.68	246.95	< 0.0001*	
Residual	18.87	15	1.26			
Lack of fit	10.81	10	1.08	0.67	0.7239	Not significant
Pure error	8.06	5	1.61			
Cor total	2937.63	29				

*Statistically significant at 95% confidence level (*p* value < 0.05)

According to the ANOVA analysis, the model *p*-value was 0.0001, which is less than 0.05, indicating that the model terms are significant. In this case, *A*, *B*, *C*, *D*, *AB*, *CD*, *A*^2^, *B*^2^, *C*^2^, and *D*^2^ are all-important model terms. The lack of fit *p*-value of 0.7239 indicates that the lack of fit is insignificant in proportion to the pure error. A non-significant lack of fit is good. The “predicted R-squared value” of 0.9748 agrees with the “adjusted R-squared value” of 0.9876. “Adequate precision” measures the signal-to-noise ratio. A ratio greater than 4 is desirable. Here, the value of 41.326 indicates an adequate signal. Thus, the model could be used to navigate the design space.

### Interaction among variables

**Fig 3:**
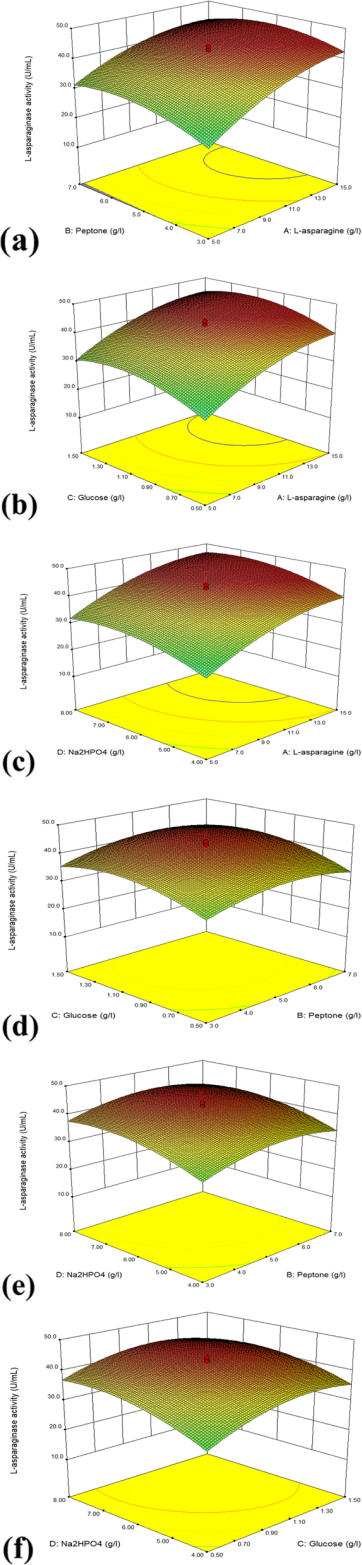
Response surface plot showing the interaction between variables **a** peptone and l-asparagine, **b** glucose and l-asparagine, **c** Na_2_HPO_4_ and l-asparagine, **d** glucose and peptone, **e** peptone and Na_2_HPO_4_, and **f** glucose and Na_2_HPO_4_

The effects of variable interactions on l-asparaginase activity were investigated. Optimized 3D surface plots were used to depict the interactive effects of any two factors. In this plot, two variables were kept constant while the other one was present in the investigation range. The level of each variable influencing the maximum yield of l-asparaginase was analyzed. The 3D response plot shown in Fig. 3a depicts the interaction between l-asparagine and peptone. The l-asparaginase activity increases with the increasing concentration of l-asparagine; the activity starts to decline only towards its maximum concentration. In the case of peptone, the enzyme activity is highest at its intermediate value and begins to decline after that. This plot showed a strong interaction between variables as indicated by *p* < 0.05. Figure 3b shows the interaction between l-asparagine and glucose. In this case, enzyme activity increases with a higher concentration of l-asparagine and starts declining at its maximal point. As the glucose concentration increases, the enzyme activity also increases up to its median value and starts declining beyond that. Because the *p*-value is so high, there was no interaction between these two factors. Figure 3c explains the interaction between l-asparagine and Na_2_HPO_4_. Here, enzyme activity increases as l-asparagine and Na_2_HPO_4_ concentrations increase, and it starts declining only at its maximum value. There was no evidence of an interaction effect between these variables. Figure 3d describes the interaction between peptone and glucose. According to the graph, the enzyme activity is highest when the concentrations of peptone and glucose are at their midpoints; activity declines beyond this point as the concentrations of variables increase. There was no interactive effect among these variables.

Figure 3e shows the interaction between peptone and Na_2_HPO_4_. The enzyme activity becomes maximum when peptone reaches its middle value, whereas in the case of Na_2_HPO_4_, the enzyme activity starts declining towards its end value. In this case, also we could not see any interaction among these variables. Figure 3f depicts the interaction between glucose and Na_2_HPO_4_. Enzyme activity increases as the glucose concentration reach its middle value and start declining beyond that. In the case of Na_2_HPO_4_, enzyme activity rises beyond the middle value and begins to decline only when it approaches its maximum value. This graph also showed the strong interaction between variables as indicated by *p* < 0.05.

### Validation of the model

To verify the adequacy of statistical analysis, some experiments were suggested by the software and were carried out in duplicates (Table 7). The maximum l-asparaginase activity predicted by the experimental design using the optimum concentrations of selected components (11.50 g/l l-asparagine, 5.30 g/l peptone, 1.32 g/l glucose, and 7.54 g/l Na_2_HPO_4_) was 45.04 ± 0.42 U/mL, and this value was in agreement with the experimental yield of 45.59 ± 0.36 U/mL. This experimental result verifies the validity of the model and the existence of optimal points.

**Table 7:** Validation of the model

Run	l-asparagine	Peptone	Glucose	Na_2_HPO_4_	l-asparaginase activity (U/mL)		Residual
	g/l	g/l	g/l	g/l	Observed	Predicted	
1	11.80	6.30	0.98	7.66	45.2431	44.6565	0.59
2	11.50	5.30	1.32	7.54	45.5903	45.0408	0.55
3	11.02	4.57	1.19	7.44	43.8542	44.5338	− 0.68

### Analysis of growth curve

**Fig. 4:**
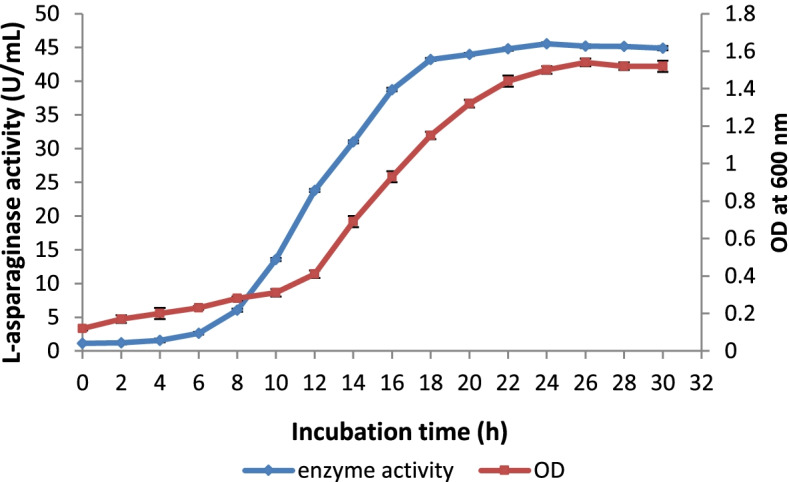
l-asparaginase production profile and growth curve of *Acinetobacter baumannii* ZAS1

A result of the growth curve analysis is depicted in Fig. 4. The organism showed maximum l-asparaginase activity (45.52 ± 0.23 U/mL) at 24 h of incubation. The organism revealed a maximum growth rate after 24 h.

## Discussion

Recent developments have heightened the need to find better sources of the l-asparaginase enzyme. The endophyte *Acinetobacter baumannii* ZAS1 showed promising l-asparaginase activity. Many scholars have discussed the differences in l-asparaginase production between microorganisms from different habitats, and the impact of cultural conditions on the amount of l-asparaginase generated [11, 12]. The physical factors and the culture media composition considerably influence the cell growth and production of metabolites [25]. The present study was performed to improve the enzyme production by this endophyte by optimizing the physical conditions and media components using response surface methodology.

Temperature is an important factor in the success of fermentation reactions. It controls the growth and production of metabolites by microorganisms and varies from one organism to another [26]. The temperature modulates extracellular enzyme synthesis by altering the physical properties of the cell membrane [27]. In the case of *Acinetobacter baumannii* ZAS1, the maximum l-asparaginase (18.854 ± 0.2 U/mL) production was observed at 37 °C, and the enzyme production was found to be sustained up to 42 °C. A gradual decline of enzyme activity was observed beyond this temperature range. The ideal temperature for the synthesis of l-asparaginase by many organisms was between 37 and 42 °C, which falls within the range of 35 to 50 °C recorded by many other researchers in their investigations. *E. coli* exhibited good production of l-asparaginase at a temperature of 37 °C [28]. According to Erva et al*.* [22], the best temperature for the enhanced production of l-asparaginase by *Bacillus subtilis* VUVD001 was 49.9 °C.

Another physical factor that has a significant role in microbial growth and metabolite production is pH. *Acinetobacter baumannii* ZAS1 showed its maximum l-asparaginase activity of 19.201 ± 0.2 U/mL at pH 7 and lower enzyme activity below and above pH 7. Similarly, Ghosh et al. [10] obtained maximum l-asparaginase activity at a neutral pH of 7.4 for *Serratia marcescens.*

Agitation speed is a critical parameter that influences enzyme production by providing adequate mixing, increasing the oxygen transfer, and maintaining the homogeneous chemical and physical conditions in the medium [29]. The endophyte *Acinetobacter baumannii* ZAS1 showed maximum l-asparaginase production of 18.854 ± 0.6 U/mL with an agitation speed of 150 rpm. Similarly, the bacteria *E. coli* ATCC 11303 [30] exhibited maximum l-asparaginase activity when incubated under an agitation speed of 150 rpm. A slightly higher agitation speed of 200 rpm was found to be effective for producing l-asparaginase by *Bacillus subtilis* VUVD001 [22]. There was a report on a very high agitation speed of 500 rpm for better production of l-asparaginase by *Erwinia aroideae* [31]*.* It shows that optimum agitation speed varies among microbes.

The preliminary screening of different nitrogen, carbon, and ion sources through the OFAT method revealed that the supplementation of these ingredients considerably enhanced enzyme production. Carbon sources are essential components in constructing cellular materials and are also used as energy sources [32]. Several earlier studies revealed the importance of additional nitrogen, carbon, and ion sources in the augmented production of this enzyme [33]. The organism *Enterobacter aerogenes* showed maximum l-asparaginase production in the presence of diammonium hydrogen phosphate and sodium citrate as nitrogen and carbon sources, respectively [34]. *Pseudomonas resinovorans* IGS-131 exhibited maximum production of l-asparaginase when Na_2_HPO_4_, KH_2_PO_4_, and NaCl were used as ion sources [35].

In the present study, screening of different media ingredients for the maximal production of l-asparaginase by *Acinetobacter baumannii* ZAS1 was performed through PBD experiments. PBD was successfully used in previous studies to optimize media components for maximum l-asparaginase activity by *Bacillus* sp. GH5 [36] as well as enhanced production of glucoamylase by *Humicola grisea* MTCC 352 [37]. This experiment identified four ingredients, namely, l-asparagine, peptone, glucose, and Na_2_HPO_4_, as statistically significant using Plackett-Burman design. The *R*^2^ score in this study was 0.9445, indicating that the model was good. Furthermore, the adjusted *R*^2^ of 0.9042 was relatively high, showing that the model was highly significant. This model had a higher predicted *R*^2^ (0.8166) value, indicating that it can predict the value of l-asparaginase synthesis in the range of parameters utilized. Similar work has been reported in PBD experiment of *Streptomyces rochei* in the improved production of l-asparaginase [38].

In this study, l-asparagine showed a significant effect with (*p* = 0) on the production of l-asparaginase. In previous investigations, the amino acid l-asparagine was discovered as a natural substrate for synthesizing l-asparaginase [39]. The second variable chosen via PBD, namely peptone, contains various amino acids and short peptides that may act as additional stimulatory components for the production of the l-asparaginase enzyme. Some previous works also reported the importance of peptone as an additional supplement for the best production of l-asparaginase by *Streptomyces ginsengisoli* [40]. In the present investigation, the carbon source glucose (simple sugar) was the third variable selected through PBD, which significantly affects the production of l-asparaginase. Reports were available to depict the importance of glucose in producing l-asparaginase from different microbes [40]. Ions also play a crucial role in the metabolic process of all organisms, as it is essential for the formation of cell mass and acts as a cofactor for many biosynthetic enzymes to catalyze the necessary reactions [41]. Borkotaky and Bezbaurauh [42] have reported no inhibitory effect on the production of l-asparaginase by 10 mmol/L metal ions such as Na^2+^, K^+^, Mg^2+^, Zn^2+^, Ca^2+^, Co^2+^, Ba^2+^, and Ni^2+^. In this study, Na_2_HPO_4_ had been selected as the significant ion source for the improved production of l-asparaginase enzyme. The optimization studies of *Bacillus sp*. GH5 through statistical analyses had revealed the significance of Na_2_HPO_4_ in the production of l-asparaginase [36].

Further optimizations of these selected factors for maximizing the l-asparaginase activity were performed through the CCD of RSM. In an analysis of variance (ANOVA), the closer the *R*^2^ number is to 1.0, the stronger the model is and the better it predicts the reaction of the system [43]. In the current study, we obtained an *R*^2^ value near 1.0, indicating the model’s strength. The ANOVA results of this study revealed the importance of a well-designed experimental model that accurately depicts the relationship of the variables in the enhanced l-asparaginase activity. The higher *F* value (165.72) in the ANOVA findings implies that the second-order model equation derived was significant. Lack of fit *F* value can also be used to confirm the importance of the second-order model. Lack of fit has a lower *F* value (0.67) than the other variables, with larger *F* values. The non-significant lack of fit value suggests that the model was significant [44]. There is a previous report on the high *F* value (43.04) of the model and lower *F* value (2.98) of lack of fit that suggested the significance of the model in the optimization of glucoamylase production by *Humicola grisea* MTCC 352 [45].

The interactive effect of the above described four variables on the l-asparaginase output by *Acinetobacter baumannii* ZAS1 was studied using this method. Response surface plots were used to illustrate the function of two variables at a time while keeping all other variables constant. As a result, these graphs were more helpful in comprehending the interaction effects of these two variables [46].

In the validation of the present study, a 2.41-fold increase in l-asparaginase activity (18.85 ± 0.2 U/mL to 45.59 ± 0.36 U/mL) by *Acinetobacter baumannii* ZAS1 has been obtained with optimized medium (11.50 g/l l-asparagine, 5.30 g/l peptone, 1.32 g/l glucose, and 7.54 g/l Na_2_HPO_4_)_._ Similarly, Kenari et al. [30], in their study, revealed that the highest l-asparaginase activity by *E. coli* ATCC 11303 was observed by optimizing the media ingredients through RSM.

The growth curve study revealed that the organism's growth and the production of l-asparaginase were associated. The most activity was seen in the late log phase, and after that, it steadily declined. Similar results were reported by Shirazian et al. [47]. According to their study, the halophilic *Bacillus strain* gA5 revealed the correlation between growth and l-asparaginase production.

## Conclusions

Based on the results of this experiment, we concluded that the endophyte *Acinetobacter baumannii* ZAS1 is the best source of the l-asparaginase enzyme. The enzyme production with this strain was enhanced by optimizing process parameters through statistical methods PBD and CCD of RSM. We were able to find the ideal parameters for obtaining the best l-asparaginase production (45.59 ± 0.36 U/mL) using these methods. Statistical optimization showed a 2.41-fold increase in enzyme production compared to unoptimized media. Finally, this research had demonstrated that experimental designs gave a quick and meaningful approach to improve productivity of l-asparaginase. The findings showed that *Acinetobacter baumannii* ZAS1 can produce large quantities of l-asparaginase with minimal medium components, implying that its usage in industries could result in significant cost reductions on an industrial scale.

## Supplementary Information

**Additional file 1: Supplementary Figure 1.** Normal plot of residuals and other plots of CCD.

## Data Availability

All data generated or analyzed during this study are included in this article.

## Abbreviations

ALL: Acute lymphoblastic leukemia
ANOVA: Analysis of variance
CCD: Central composite design
OFAT: One factor at a time
PBD: Plackett–Burman design
RSM: Response surface methodology
UGC: University Grants Commission
