# Supplementary figures and images for: Response surface methodological optimization of l-asparaginase production from the medicinal plant endophyte Acinetobacter baumannii ZAS1
Source: J Genet Eng Biotechnol. 2022 Feb 9;20:22. doi: 10.1186/s43141-022-00309-4 (PMC8828825; doi:10.1186/s43141-022-00309-4)

**Supplementary data**

| **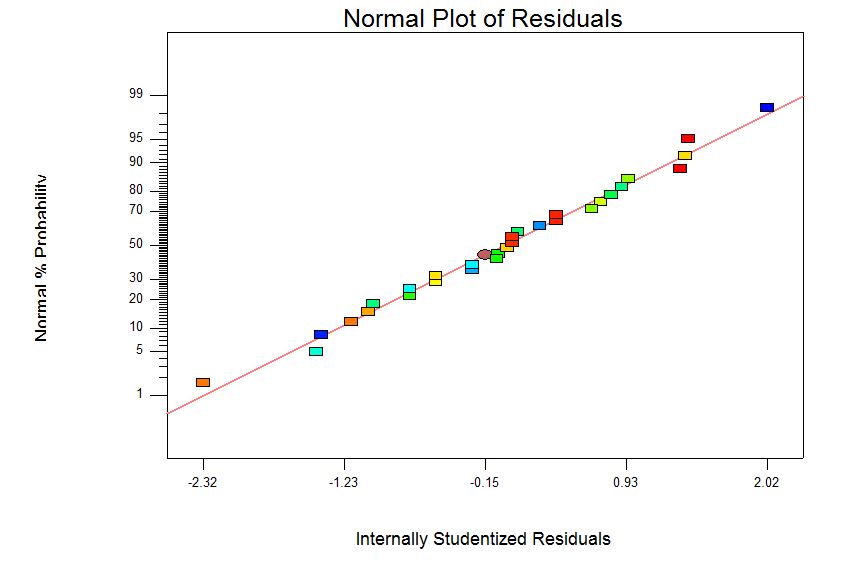** |
| --- |
| **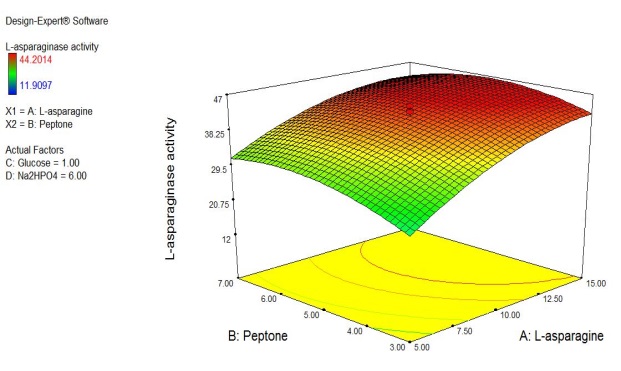** |
| **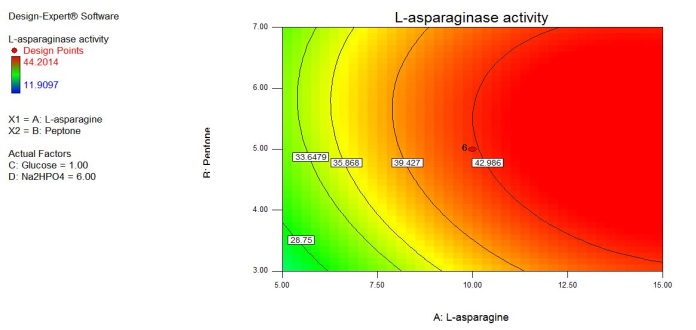** |
| **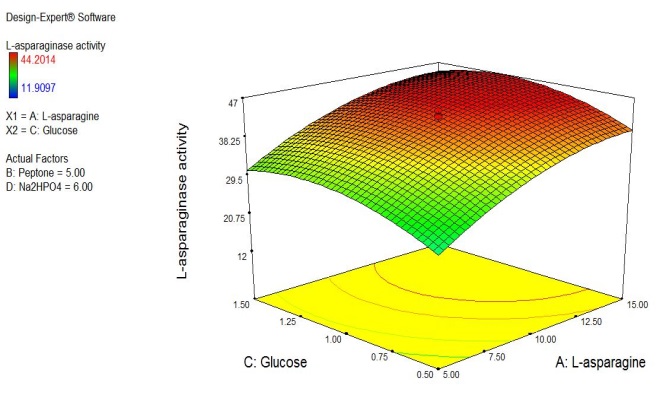** |
| **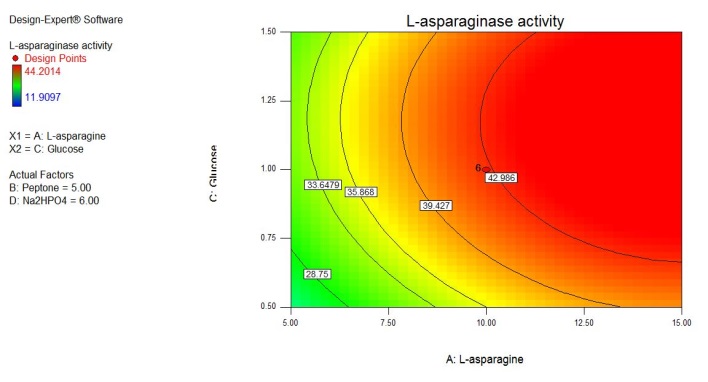** |
| **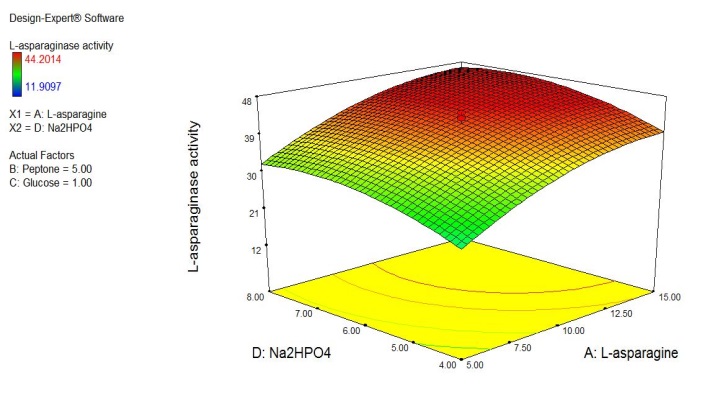** |
| **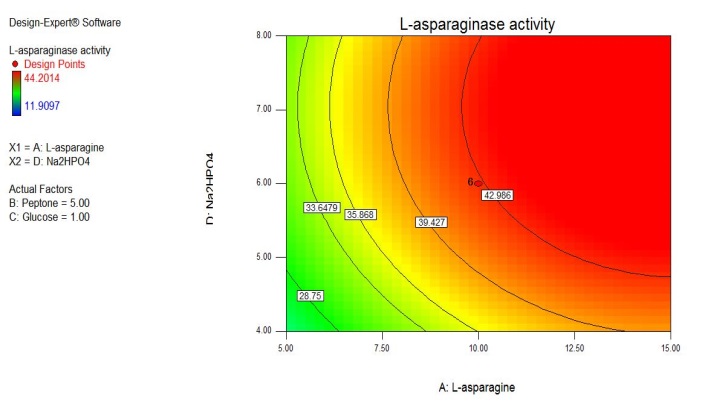** |
| **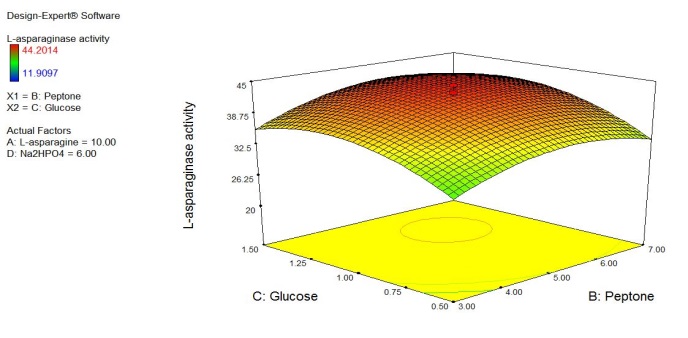** |
| **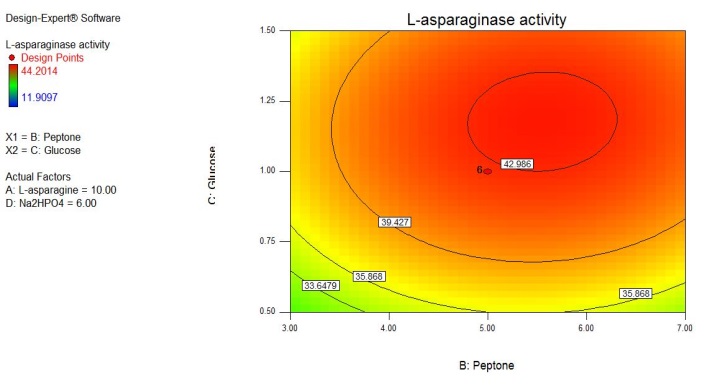** |
| **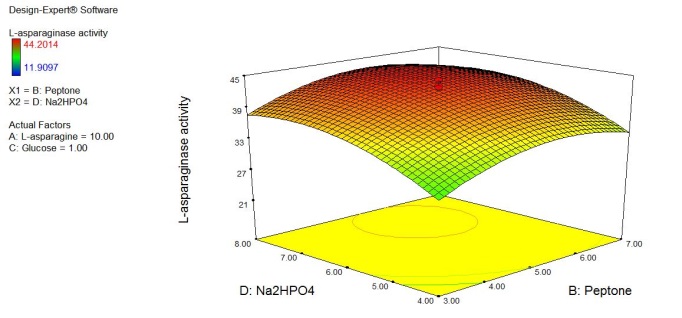** |
| **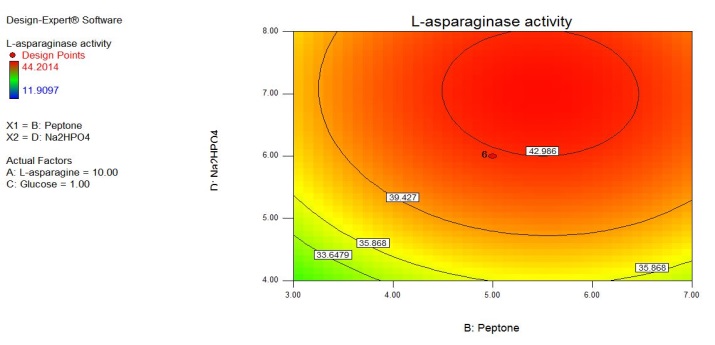** |
| **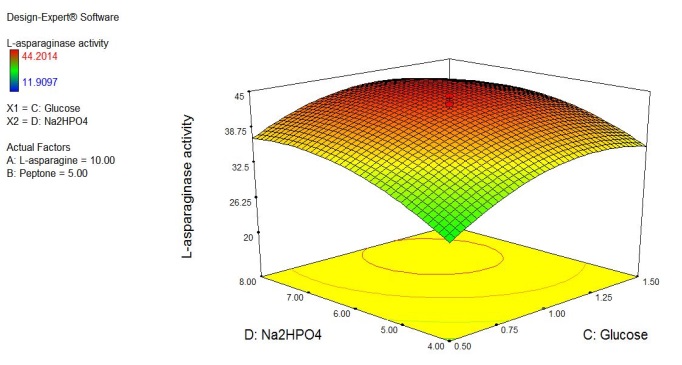** |
| **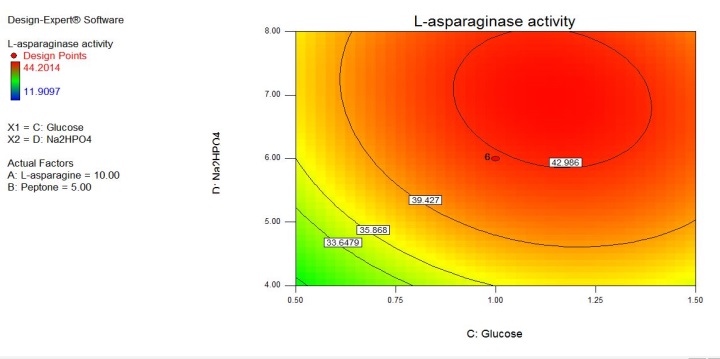** |

**Supplementary Figure 1.** Normal plot of residuals and other plots of CCD

Supplement: Supplementary file 1 — Additional file 1: Supplementary Figure 1. Normal plot of residuals and other plots of CCD. [file 43141_2022_309_MOESM1_ESM.docx]
